# Artificial Intelligence-Based Conversational Agents for Chronic Conditions: Systematic Literature Review

**DOI:** 10.2196/20701

**Published:** 2020-09-14

**Authors:** Theresa Schachner, Roman Keller, Florian v Wangenheim

**Affiliations:** 1 Department of Management, Technology, and Economics ETH Zurich Zurich Switzerland; 2 Future Health Technologies programme Campus for Research Excellence and Technological Enterprise Singapore-ETH Centre Singapore

**Keywords:** artificial intelligence, conversational agents, chatbots, healthcare, chronic diseases, systematic literature review

## Abstract

**Background:**

A rising number of conversational agents or chatbots are equipped with artificial intelligence (AI) architecture. They are increasingly prevalent in health care applications such as those providing education and support to patients with chronic diseases, one of the leading causes of death in the 21st century. AI-based chatbots enable more effective and frequent interactions with such patients.

**Objective:**

The goal of this systematic literature review is to review the characteristics, health care conditions, and AI architectures of AI-based conversational agents designed specifically for chronic diseases.

**Methods:**

We conducted a systematic literature review using PubMed MEDLINE, EMBASE, PyscInfo, CINAHL, ACM Digital Library, ScienceDirect, and Web of Science. We applied a predefined search strategy using the terms “conversational agent,” “healthcare,” “artificial intelligence,” and their synonyms. We updated the search results using Google alerts, and screened reference lists for other relevant articles. We included primary research studies that involved the prevention, treatment, or rehabilitation of chronic diseases, involved a conversational agent, and included any kind of AI architecture. Two independent reviewers conducted screening and data extraction, and Cohen kappa was used to measure interrater agreement.A narrative approach was applied for data synthesis.

**Results:**

The literature search found 2052 articles, out of which 10 papers met the inclusion criteria. The small number of identified studies together with the prevalence of quasi-experimental studies (n=7) and prevailing prototype nature of the chatbots (n=7) revealed the immaturity of the field. The reported chatbots addressed a broad variety of chronic diseases (n=6), showcasing a tendency to develop specialized conversational agents for individual chronic conditions. However, there lacks comparison of these chatbots within and between chronic diseases. In addition, the reported evaluation measures were not standardized, and the addressed health goals showed a large range. Together, these study characteristics complicated comparability and open room for future research. While natural language processing represented the most used AI technique (n=7) and the majority of conversational agents allowed for multimodal interaction (n=6), the identified studies demonstrated broad heterogeneity, lack of depth of reported AI techniques and systems, and inconsistent usage of taxonomy of the underlying AI software, further aggravating comparability and generalizability of study results.

**Conclusions:**

The literature on AI-based conversational agents for chronic conditions is scarce and mostly consists of quasi-experimental studies with chatbots in prototype stage that use natural language processing and allow for multimodal user interaction. Future research could profit from evidence-based evaluation of the AI-based conversational agents and comparison thereof within and between different chronic health conditions. Besides increased comparability, the quality of chatbots developed for specific chronic conditions and their subsequent impact on the target patients could be enhanced by more structured development and standardized evaluation processes.

## Introduction

Conversational agents or chatbots are computer systems that imitate natural conversation with human users through images and written or spoken language [1]. This paper focuses on conversational agents that deploy intelligent software or artificial intelligence (AI), which is increasingly used for applications in credit scoring [2], marketing strategies [3], and medical image analysis in radiology [4].

There are several ways of defining AI, as discussed by Russel and Norvig [5] in 1995. Their commonality is that AI describes algorithms that artificially emulate human cognitive and behavioral thought processes and are instantiated in software programs. Since then, the number of definitions had risen with the growing number of AI applications [6]. There are several specific understandings of AI such as by De Bruyn et al [7], who define AI as software that can “autonomously generate new constructs and knowledge structures” [7]. More general approaches describe and distinguish between weak AI, strong AI, and artificial general intelligence (AGI). Coined by John Searle in 1980, the term weak AI describes software that appears intelligent by mimicking specific human cognitive processes such as image recognition or natural language processing [8]. Strong AI denotes software that truly possesses intelligence without mimicking it [8]. AGI as an expansion of these terms designates true intelligence for all human cognitive processes instead of just for individual tasks [9,10]. For this paper, we adopt the understanding of weak AI when talking about AI-based conversational agents; the algorithms implemented in the conversational agent software each mimic distinct and narrowly restricted human cognitive processes.

The latest advances in AI allow for increasingly natural interactions between humans and their machine agent counterparts [11,12]. This emulated human-machine communication becomes more complex and sophisticated, especially through advancements in machine learning with the application of neural networks [13-15]. This is reflected in the rising number of conversational agents that aim at human-like exchanges [16] in fields such as e-commerce, travel, tourism, and health care [17-19]. Well-known examples of such intelligent chatbots are Microsoft’s Cortana, Amazon’s Alexa, or Apple’s Siri [12].

The focus on the human-machine relationship was present from the very beginning in the history of chatbots; the rule-based software program ELIZA [20] was designed to take on the role of a psychotherapist in order to mimic a patient-centered Rogerian psychotherapy exchange. Developed in 1966 by Joseph Weizenbaum, it was then followed by PARRY, another mental health care–related chatbot developed in 1972 [21]. While ELIZA played the role of the therapist, PARRY took on the part of a schizophrenic patient [20,21]. Even though ELIZA passed a restricted Turing Test—a machine intelligence test with the success criterion of whether a human can distinguish a machine from a human during a conversation [22]—it was a rule-based and pre-scripted software program [23]. Similarly, other early forms of the then-called chatterbots such as Psyxpert, an expert system for disease diagnosis support written in Prolog [24] or SESAM-DIABETE, an expert system for diabetic patient education written in Lisp [25], followed a rule-based approach. ALICE (Artificial Linguistic Internet Computer Entity), in 1995, was the first computer system to use natural language processing for the interpretation of user input [12].

Since then, increasingly efficient access to and storage of data, decreasing hardware costs, and eased access to cloud-based services improved the development of AI architecture [26]. These advances gave rise to a more standardized deployment of natural language processing, voice recognition, natural language generation, and the like within chatbot development [11,12].

In health care, such AI-based conversational agents have demonstrated multiple benefits for disease diagnosis, monitoring, or treatment support in the last two decades [1,19,27,28]. They are used as digital interventions to deliver cost-efficient, scalable, and personalized medical support solutions that can be delivered at any time and any place via web-based or mobile apps [29-31]. Research studies have investigated a variety of AI-based conversational agents for different health care applications such as providing information to breast cancer patients [32]; providing information about sex, drugs, and alcohol to adolescents [33]; self-anamnesis for therapy patients [34]; assistance for health coaching to promote a healthy lifestyle [35]; or smoking cessation [36].

This paper focuses on one of the most urgent health care challenges of the 21st century—the rise of chronic conditions [37]. Chronic diseases are one of the leading drivers for reduced quality of life and increased economic health care expenses through repeated hospitalization, disability, and treatment expenditures [38]. In the United States alone, they affected over 50% of adults in 2016 and accounted for 86% of health care spending [37]. Hvidberg et al [39] and others defined chronic conditions as ailments that are anticipated to last at least 12 or more months, lead to functional limitations, and require continuous medical support [40,41]. As such, they require fundamentally different prevention, treatment, and management approaches than acute conditions, which are episodic, allow for general solutions, and can be treated within health care sites [37]. In contrast, chronic conditions require challenging lifestyle and behavioral changes, frequent self-care, and ongoing and personalized treatment that go beyond traditional health care sites and reach personal settings [37,42,43]. AI-based conversational agents provide suitable, personalized, and affordable digital solutions to react to these challenges and slow down individual disease deterioration to delay premature death.

Systematic literature reviews investigated a variety of contexts of health care chatbots such as the role of conversational agents in health care in general [1] and in mental health [44], aspects of personalization of health care chatbots [45], as well as technical aspects of AI systems and architectures of conversational agents in health care [11]. However, there is surprisingly little systematic information on the application of AI-based conversational agents in health care for chronic diseases. This paper closes the gap. The objective of this paper is to identify the state of research of AI-based conversational agents in health care for chronic diseases. We extract stable findings and structures by outlining conversational agent characteristics, their underlying AI architectures, and health care applications. Additionally, we outline gaps and important open points that serve as guidelines for future research.

## Methods

### Reporting Standards

We performed a systematic literature review and followed the Preferred Reporting Items for Systematic Reviews and Meta-Analyses (PRISMA) checklist [46]. The review protocol is available in the Multimedia Appendix 1.

### Search Strategy

The search was conducted electronically during February 2020, using PubMed MEDLINE, EMBASE, PyscInfo, CINAHL, ACM Digital Library, ScienceDirect, and Web of Science. These databases were chosen as they cover relevant aspects in medicine and technology and have been used in other systematic literature reviews covering similar topics [1,45]. The search was updated by additional abstracts retrieved through various Google alerts covering different combinations of the search term until April 2020. The reference lists of other relevant literature reviews and articles were screened for additional articles. The process of query construction was initially informed by the first author’s experience in the investigated areas and extended by incorporating associated terms such as synonyms, acronyms, and commonly known terms of the same context. The final search term included an extensive list of items describing the constructs “conversational agent,” “healthcare,” and “artificial intelligence” to ensure exhaustive coverage of the search space. The complete overview of the search terms for each construct is available in Multimedia Appendix 2. An exemplary search strategy is shown for PubMed MEDLINE in Table 1.

**Table 1:** The search strategy used in PubMed MEDLINE.

Search category	Search terms
Health care	“healthcare” OR “digital healthcare” OR “digital health” OR “health” OR “mobile health” OR “mHealth” OR “mobile healthcare”
Conversational agents	“conversational agent” OR “conversational agents” OR “conversational system” OR “conversational systems” OR “dialog system” OR “dialog systems” OR “dialogue systems” OR “dialogue system” OR “assistance technology” OR “assistance technologies” OR “relational agent” OR “relational agents” OR “chatbot” OR “chatbots” OR “digital agent” OR “digital agents” OR “digital assistant” OR “digital assistants” OR “virtual assistant” OR “virtual assistants”
Artificial intelligence	“artificial intelligence” OR “AI” OR “natural language processing” OR “NLP” OR “natural language understanding” OR “NLU” OR “machine learning” OR “deep learning” OR “neural network” OR “neural networks”
Combined	1 AND 2 AND 3

### Selection Criteria

We included studies if they (1) were primary research studies that involved the prevention, treatment, or rehabilitation of chronic diseases; (2) involved a conversational agent; and (3) included any kind of artificial intelligence technique such as natural language understanding or deep learning for data processing.

Articles were excluded if they (1) involved only non-AI software architecture; (2) involved purely Wizard of Oz–based studies where the dialogue between human and conversational agent was mimicked by a human rather than performed by the conversational agent; (3) addressed health conditions and diseases that cannot conclusively be referred to as chronic diseases, general health, or any form of prechronic health conditions such as general well-being for the prevention of chronic diseases; (4) addressed chronic health conditions on a general level without specifying a disease or if the chronic disease only played a minor role for the study or was only mentioned in a few sentences.

Furthermore, we excluded studies without specific applications of conversational agents or where the application of the conversational agent for chronic diseases was only mentioned as a possibility or in a couple of sentences. We also excluded non-English papers, conference papers, workshop papers, literature reviews, posters, PowerPoint presentations, articles presented at doctoral colloquia, or if the article’s full text was not accessible for the study authors.

### Selection Process

All references that were identified through the searches were downloaded into Excel (Microsoft Corporation) and inserted in an Excel spreadsheet. Duplicates were removed. Screening was conducted by two independent reviewers in three phases, assessing first the article titles, followed by the abstracts, and finally the full texts. After each of these phases, Cohen kappa was calculated to measure interrater reliability between the researchers and determine the level of agreement [47]. Any disagreements were discussed and resolved in consensus.

### Data Extraction

The two reviewers familiarized themselves with the identified articles and then independently extracted the contained information into an Excel spreadsheet with 30 columns containing information on the following aspects: (1) general information about the included studies, (2) health care/chronic conditions, (3) conversational agents, (4) AI, and (5) additional study items such as conflict of interests or reported funding. We extracted data such as first author, year of publication, study design/type, study aim, conversational agent evaluation measures, main reported outcomes and findings, type of chronic condition, type of study participants, AI technique, AI system development, sources of funding, and conflicts of interest.

The full list can be seen in Multimedia Appendix 3. The extracted data were synthesized narratively. Quality of studies was not assessed in this analysis due to the diversity of analyzed studies. Any inconsistencies after the individual data extractions were discussed and resolved in consensus agreement.

### Risk of Methodological Bias

The author team engaged in extensive discussion about the selection of an appropriate tool to assess methodological biases of the included studies, given the variety of study designs and the diversity of reported evaluation measures.

After extensive research in relevant journals, we decided to follow the approach of Maher et al [48], who devised a risk assessment tool based on the Consolidated Standards of Reporting Trials (CONSORT) checklist [49]. The tool developed by Maher et al [48] contains all 25 items from the CONSORT checklist and assigns scores of 1 or 0 to each item per study, indicating whether the item was satisfactorily fulfilled or not in the respective study. Lower scores imply higher risk of methodological bias and the inverse for higher scores. Whereas the CONSORT checklist was originally developed for controlled trials, we concluded that most of its criteria are applicable. We adapted the tool by Maher et al [48] by allowing scoring from 0 to 1 in order to more precisely assess the achieved score of each checklist item per study.

The authors independently familiarized themselves with the assessment tool and rated each study individually. Cohen kappa was calculated to assess interrater reliability between the two assessments and scored at 79%; the majority of disagreement concerned generalizability and sample size guidelines. Discrepancies were discussed and resolved in consensus. For details on the risk bias tool used and the authors’ ratings, see Multimedia Appendix 4.

## Results

### Selection and Inclusion of Studies

**Figure 1:**
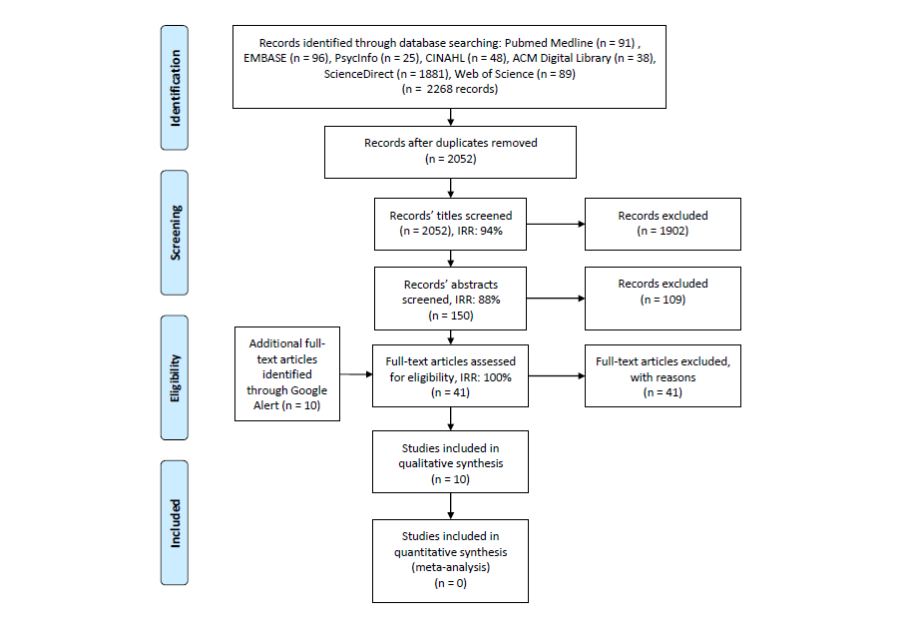
Preferred Reporting Items for Systematic Reviews and Meta-Analyses flow diagram of included studies. Search updates were conducted until April 2020, with no additional papers being identified for inclusion. IRR: interrater reliability.

In all, 2052 deduplicated citations from electronic databases were screened (Figure 1). Of these, 1902 papers were excluded during the title and abstract screening processes, respectively, leaving 41 papers eligible for full-text screening. The search was updated at full-text stage by 10 additional papers identified through Google Alerts, making 51 papers eligible for full-text screening. On reading the full texts, 41 papers were found to be ineligible for study inclusion. Ultimately, 10 papers were considered eligible for inclusion into our systematic literature review.

### Characteristics of Included Studies

The full list of included studies can be seen in Table 2. Article publication dates ranged from 2010 to 2020, with 80% (8/10) papers published from 2016 onward. Four studies were conducted in the United States [50-53], 2 in Spain [54,55], and 1 each in Australia [56], Canada [57], United Kingdom [58], and Korea [59]. Most studies were quasi-experimental and involved users testing and evaluating the conversational agents [50,51,54,56-59]. Two studies were randomized controlled trials (RCTs) [52,53], and 1 was a proof-of-concept study [55].

Of the 10 studies, 4 aimed to design, develop, or evaluate a prototype conversational agent [50,51,58,59]. One study aimed to develop and implement a prototype architecture of a conversational agent [55]. Three studies aimed to only evaluate a specific conversational agent [52,53,56], and 1 study aimed to design, implement, and evaluate a specific conversational agent [57]. One study aimed to design and develop a domain-independent framework for the development of conversational agents and evaluate a corresponding prototype [54].

Three of 10 studies did not report on the sources of funding [54,56,57]. Seven studies reported no conflict of interest [50,51,54,55,57-59]. Two studies disclosed a relevant conflict of interest (see Multimedia Appendix 3) [52,53], and 1 study did not report upon conflict of interests [56].

**Table 2:** Overview and characteristics of included studies.

Study ID, study location, study design	Study aim	Main reported outcomes and findings	Type and number of study participants	Chronic condition addressed	Type of final target interaction recipient	Health/ application goal
Ferguson et al (2010), US, quasi-experimental	Design and development of prototype system	Prototype development for data collection, sufficient user engagement, development of working end-to-end spoken dialogue system for heart failure check-up	Heart failure patients (focus group: n= 9; survey: n=63)	Heart failure	Patients	Self-care support
Rhee et al (2014), US, quasi-experimental	Design and development of prototype system	High response rate for daily messages of adolescents (81%-97%), symptoms most common topic in adolescent-initiated messages, improvement of symptom and trigger awareness, promoted treatment adherence and sense of control, facilitation of adolescent-parent partnership	Adolescent asthma patient-parent dyads (n=15)	Asthma	Patient-parent dyads	Self-management tool
Griol and Callejas (2016), Spain, quasi-experimental	Design, development, and evaluation of domain-independent framework	Patient feedback: satisfactory system interaction, preference for multimodal interaction due to flexibility; caregiver feedback: positive assessment, perceived potential to stimulate cognitive abilities of patients	Alzheimer patients (n=25) and caregivers (n=6)	Alzheimer	Patients	Disease monitoring
Ireland et al (2016), Australia, quasi-experimental	Evaluation of chatbot	Positive overall impression, technical issues with speed of processing	Community members (n=33)	Parkinson/dementia	Patients	General conversation with Parkinson patients and facilitation of assessments; future: speech and communication therapy for patients
Fitzpatrick et al (2017), US, RCT^a^	Evaluation of fully automated conversational agent	Chatbot interaction significantly reduced depression and associated with high level of engagement and viewed as more favorable than information-only control comparison	Students (n=70)	Depression/anxiety	NA^b^	CBT^c^
Fulmer et al (2018), US, RCT	Evaluation of fully automated conversational agent	2 weeks of chatbot interaction with daily check-ins significantly reduced symptoms of depression, 4 weeks of chatbot interaction reduced symptoms of anxiety more than 2 weeks of chatbot interaction, chatbot interaction led to higher engagement and higher overall satisfaction than control intervention	Students (n=74)	Depression/anxiety	NA	Health support via different interventions such as CBT, mindfulness-based therapy
Easton et al (2019), UK, quasi-experimental	Co-design of prototype and acceptability assessment	Specification of 4 distinct self-management scenarios for patient support, positive engagement, AI^d^-based speech recognition did not work sufficiently - replacement with human wizard for video-based scenario testing	Co-design: COPD^e^ patients (n=6), health professionals (n=5), video-based scenario testing: COPD patients (n=12)	COPD	Patients	Self-management tool
Rose-Davis et al (2019), Canada, quasi-experimental	Design, implementation, and evaluation of prototype dialogue system	Implementation of AI-based extended model of argument into conversational agent prototype for delivering patient education, satisfactory feedback	Clinicians (n=6)	JIA^f^	Parents of patients	Patient education
Roca et al (2020), Spain, proof of concept	Development and prototype architecture implementation of chatbot	Development of prototype chatbot architecture based on microservices through the use of messaging platforms	Health care professionals (n=NA)	Variety of chronic diseases, specific example of psoriasis	Patients	Disease monitoring
Rehman et al (2020), Korea, quasi-experimental	Design, development, and evaluation of prototype chatbot	Algorithm performance: accuracy: 89%, precision: 90%, sensitivity: 89.9%, specificity: 94.9%, F-measure: 89.9%, good results in all user experience aspects, efficient disease prediction based on chief complaints	Students (n=33)	Diabetes, glaucoma	Patients	Disease diagnosis

^a^RCT: randomized controlled trial.

^b^NA: not available.

^c^CBT: cognitive behavioral therapy.

^d^AI: artificial intelligence.

^e^COPD: chronic obstructive pulmonary disease.

^f^JIA: juvenile idiopathic arthritis.

### Evaluation Measures and Main Findings

Two studies assessed the technical performance of the conversational agents and reported consistently high performance measures of the conversational agent (accuracy: 89%; precision: 90%; sensitivity: 89.9%; specificity: 94.9%; F-measure: 89.9%) [59] as well as high message response rates (81% to 97%) [51].

In 7 studies, user experience was assessed. User experience was generally positive regarding the acceptability, understanding of the conversational agents, comprehensibility of the systems’ responses, interaction rates, or content relevance [51,53,54,56-59].

Two RCTs reported on health-related outcomes and found that interaction with the conversational agents led to decreased symptoms of depression and anxiety compared with the control groups [52,53].

Four studies found high levels of engagement with the conversational agent or reported the conversational agent to be engaging [50,52,53,58]. One study found that the conversational agent improved awareness of disease symptoms and triggered and promoted treatment adherence [51].

One study reported that the developed conversational agent architecture was able to provide telemonitoring for chronic diseases [55]. The same study further received feedback of health professionals that the architecture provides a flexible solution for personalized monitoring services and data storage [55].

### Health Care Characteristics

In the reviewed articles, psychological conditions were the most commonly addressed type of condition, which was the focus of 3 studies [52-54]. Other types of chronic conditions included respiratory [51,58], cardiovascular [50], nervous system [56], rheumatic [57], and autoimmune/eye conditions [59]. One study addressed various chronic diseases and outlined a specific example of an autoimmune disease [55]. More specifically, the addressed chronic conditions included depression and anxiety [52,53], heart failure [50], asthma [51], Alzheimer disease [54], Parkinson/dementia [56], chronic obstructive pulmonary disease (COPD) [58], juvenile idiopathic arthritis (JIA) [57], and diabetes/glaucoma [59]. One study addressed a variety of chronic diseases and delineated psoriasis as a specific example [55].

In 3 papers, students served as main study participants [52,53,59]. Disease-specific patients were involved in 3 studies [50,54,58]. Other types of study participants included patients’ parents [51], caregivers [54], clinicians [57], health professionals [55,58], and community members [56].

Patients were the most common final targeted interaction recipients [50,54-56,58,59]. One study targeted the interaction for the use with patient-parent dyads [51], whereas 1 other study specifically targeted patients’ parents [57]. Two studies did not provide further information on the targeted interaction recipients [52,53].

Self-care and self-management were the main health goals of the conversational agents in 3 studies [50,51,58], whereas 2 study agents were sought to assist in disease monitoring [54,55]. Other study health goals included general conversations with patients [56], cognitive behavioral therapy [52], patient education [57], and disease diagnosis [59]. One study reported health support via different interventions such as cognitive behavioral or mindfulness-based therapy [53].

Of the 10 studies, 2 aimed at further human involvement besides the targeted interaction recipients. One study additionally involved patients’ parents as well as a certified asthma expert [51], and another study involved patients’ caregivers [57].

### Characteristics of Conversational Agents

Conversational agents were mostly used for data collection [50,54], coaching [52,53], diagnosis [55,59], and support [51,58] (see Table 3 for overview and characteristics of the conversational agents reported in the included studies). Education was the goal of one conversational agent [57] whereas another agent is currently built for data collection but it was anticipated that it may also have an educational and support purpose in future [56].

Different communication channels were used across the identified conversational agents. While two conversational agents use a smartphone app as their main communication channel [54,56], one study reports the general use of the mobile phone [51]. One agent uses a platform agnostic smartphone and desktop instant messenger app [52], and another agent uses a platform-specific application for Android and is usable on any smart Android device such as smartwatch, smartphone, tablet, laptop, and vendor-specific devices that contain a microphone and speaker and support Android [59]. Another agent employs a customizable platform that can be accessed via multiple communication channels such as Facebook, Slack, or short messaging services [53]. One agent uses a web browser as the main communication channel [58], while another agent is designed for communication channels such as messaging platforms or web interfaces [55]. The communication channel of two conversational agents was not specified in the papers [50,57].

The dialogue initiative of 4 conversational agents was held by the user [54,55,57,59], whereas 4 conversational agents used a mixed approach which means that both the user and the system were able to initiate the conversation [50-52,56]. Two studies did not report upon the dialogue initiative [53,58].

A total of 6 studies used a multimodal interaction modality which means that multiple different modalities for input and/or for output were used. Of these, 2 conversational agents require a spoken input format [56,59], whereas 2 other agents allow for both spoken or written input formats [50,58]. One conversational agent uses a written or a visual input format [55], and 1 study employs spoken, written, visual as well as external content from a smartphone sensor as an input format [54]. Regarding the output formats of the multimodal agents, 2 agents use spoken and written output formats [50,56]. One conversational agent uses only a written output format [55], whereas 1 agent employs a written or a visual output format [59]. One agent uses a spoken, written, or a visual output format [54], while 1 study did not report upon the output format used [58]. The remaining 4 studies use a written format of interaction modality, which means that both input and output were in a written form [51-53,57].

Most of the conversational agents we identified were still in a prototype stage and were not publicly available [50,51,54,55,57-59]. Two conversational agents were commercially available [52,53], and 1 was available for free on Android app store [56].

**Table 3:** Overview and characteristics of the conversational agents reported in the included studies.

Study ID	Conversational agent name	Conversational agent goal	Interaction modality (input/output format)	Availability of conversational agent	AI^a^ techniques	AI system development
Ferguson et al (2010)	Personal health management assistant	Data collection	Multimodal (s^b^ or w^c^/s or w)	NA^d^ (prototype)	Speech recognition, NLP^e^	Internal
Rhee et al (2014)	mASMAA (mobile phone-based asthma self-management aid for adolescents)	Support	Written	NA (prototype)	NLP	Internal
Griol and Callejas (2016)	NA (application, conversational agent)	Data collection	Multimodal (s, w, v^f^, external sensors/s, w, v)	NA (prototype)	NN^g^, ML^h^, ASR^i^, NLU^j^, NLG^k^, TTS^l^	External (Google API^m^)
Ireland et al (2016)	Harlie (Human and Robot Language Interaction Experiment)	Now: data collection; future: education and support	Multimodal (s/s, w)	For free on Android app store	Speech recognition incl. STT^n^ and TTS, NLP, AIML^o^	External (Google API)
Fitzpatrick et al (2017)	Woebot	Coaching	Written	Commercially available	Decision tree, NLP	External (Woebot Labs Inc)
Fulmer et al (2018)	Tess	Coaching	Written	Commercially available	Emotion algorithms, ML, NLP	External (X2AI Inc)
Easton et al (2019)	Avachat (=avatar & chat)/Ava	Support	Multimodal (s, w/NA)	NA (prototype)	Speech recognition	External (Kaldi toolkit)
Rose-Davis et al (2019)	JADE (Juvenile idiopathic Arthritis Dialogue-based Education)	Education	Written	NA (prototype)	NA	Internal
Roca et al (2020)	NA (Virtual Assistant)	Diagnosis	Multimodal (w, v/w)	NA (prototype)	AIML, NLP	NA
Rehman et al (2020)	MIRA (Medical Instructed Real-Time Assistant)	Diagnosis	Multimodal (s/w, v)	NA (prototype)	Speech recognition, NLP, NLU, NN, ML, DL^p^	Internal

^a^AI: artificial intelligence.

^b^s: spoken.

^c^w: written.

^d^NA: not available.

^e^NLP: natural language processing.

^f^v: visual.

^g^NN: neural network.

^h^ML: machine learning.

^i^ASR: automatic speech recognition.

^j^NLU: natural language understanding.

^k^NLG: natural language generation.

^l^TTS: text-to-speech.

^m^API: application programming interface.

^n^STT: speech-to-text.

^o^AIML: artificial intelligence markup language.

^p^DL: deep learning.

### Artificial Intelligence Characteristics

Natural language processing represented the most used technique [50-53,55,56,59] before speech recognition (including speech-to-text and text-to-speech) [50,54,56,58,59], machine learning [53,54,59], natural language understanding [54,59], neural networks [54,59] and artificial intelligence markup language [56,57], as shown in Table 3. The following techniques were used in one study each: deep learning [59], natural language generation [54], emotion algorithms [53], and decision trees [52]. One study used AI-based argument theory for modeling its dialogue system [57]. Additional details regarding the artificial intelligence architecture can be found in Multimedia Appendix 3.

A total of 4 studies developed the artificial intelligence system internally [50,51,57,59], and 5 studies relied on external sources [52-54,56,58]. Of the studies using external artificial intelligence systems for speech recognition (including text-to-speech and speech-to-text), 2 studies used an external Google application programming interface [54,56], and 1 study used the open-source Kaldi toolkit [58]. One study relied on the existing The Rochester Interactive Planning System natural dialogue system [51], and 1 study did not report upon the artificial intelligence system development [55].

Artificial intelligence categorization varied in its terminology across the studies. Four studies were classified as AI [53,56-58]. Other categorizations were natural interaction [50], state-of-the-art natural language understanding technology [51], fully automated [52], smart [55] and state of the art real-time assistant [59]. One study did not provide an explicit categorization [54].

## Discussion

### Principal Findings

Our systematic literature review identified 10 studies, of which 2 were RCTs and the majority were quasi-experimental studies. This is, to our knowledge, the only systematic literature review focusing specifically on AI-based conversational agents used in the context of health care for chronic diseases. Other recent reviews focused on conversational agents for either a specific health condition such as mental health [44], the general application of chatbots in health care [1], or specific features thereof such as personalization [45] or technical architectures [11].

A total of 80% of the papers that we identified were published relatively recently, from 2016 onward. Together with the small number of identified studies, this shows the immaturity of the field of AI-based conversational agents for chronic diseases. This finding is coherent with other recent reviews which found the general application of conversational agents in health care to be at a nascent but developing stage [1,11,45]. Most of the AI-based conversational agents we identified were still in a prototype stage and not publicly available. They are used for data collection, coaching, diagnosis, support, and education of patients suffering from chronic diseases.

Recent advances in AI software allow an increasing number of conversational agents to offer natural interactions between humans and their machine agent counterparts [11,12]. However, drawbacks such as biased and opaque decision-making leading to limited trust in the final outcomes still exist and are only partially solved [60]. Combined with the functional difficulty of needing large datasets for algorithmic training, this could explain the overall small number of existing applications [61].

The current chatbots operate on a variety of communication channels, out of which some are vendor specific such as tailored for Android devices. We advise future studies to keep track of such platform-dependent developments as it could point to a stronger influence of or dependence on technology providers regarding health care–related applications.

The identified research was not truly geographically diverse; 50% of studies were conducted in North America, only one each in Australia and an Asian country, and the remaining 30% in Europe. There was not a single study conducted in Africa. Additionally, 90% of these research locations are embedded in Western cultures, exerting a strong bias on the generalizability of their results. Given the worldwide prevalence of chronic conditions [37] and the need to apply health care system-specific solutions [62], future research should strive to include diverse geographies to ensure context-specific relevance. We advise to extend research foci beyond the Western socioeconomic cultural context and additionally include emerging economies such as India and China to increase variability and generalizability.

The majority of the identified studies aimed at fully designing, developing, or evaluating a conversational agent specific for only one chronic condition. This finding suggests that AI-based conversational agents evolve into providing tailored support for specific chronic conditions rather than general interventions applicable to a broad range of chronic diseases. Future research could investigate the effects of such specialization on treatment-related measures such as patient satisfaction or treatment adherence.

The evaluation measures of the identified AI-based conversational agents and their effects on the targeted chronic conditions were broad and not unified. The most commonly reported measurements were user experience and chatbot engagement, which are generalistic usability measurements for technical systems [63]. Only 2 studies assessed the technical performance of the conversational agents and 2 other studies reported on the health-related outcomes. Generally, however, the measured and reported results were positive and indicated both high overall performance and satisfactory user experience, high engagement, and positive health-related outcomes. Future research could enforce following standard guidelines for research in the health care area such as the Consolidated Standards of Reporting Trials of electronic and mobile health apps and online telehealth (CONSORT-EHEALTH) [64], the mobile health evidence reporting and assessment (mERA) checklist [65], or the Transparent Reporting of Evaluations with Nonrandomized Designs (TREND) statement [66] to increase quality and comparability of studies. The primarily quasi-experimental nature and subsequent inconsistency of evaluated measures of the found literature could explain the lack of use of such reporting guidelines at present.

Our review shows that current AI-based conversational agents address a broad variety of chronic diseases, categorized as chronic respiratory, cardiovascular, nervous system-related, rheumatic, autoimmune-related, eye-related, and psychological conditions. While it is informative to have such a wide investigation of different disease types, this variation complicates the comparability within and between conditions. Future research could aim at first developing and evaluating within-chronic disease-related differences of AI-based conversational agents (eg, individual chatbots for asthma, COPD, and sleep apnea as examples of chronic respiratory diseases) before extending their scope of research to between-chronic disease-related comparisons (eg, respiratory vs cardiovascular chronic conditions).

Following such a research agenda could lead to the development of more consistent studies with higher standards and increased validity of reported findings. Similar considerations concern the large variety of reported health goals; while self-care management is the main health goal of 30% of existing AI-based conversational agents for chronic conditions before offering assistance of disease monitoring, the remaining 70% address intervention goals such as general conversation, therapy, education, and diagnosis. This inconsistency presents another complication of the comparability of the existing chatbots.

Of the studies investigated, 70% were quasi-experimental, 20% RCTs, and the remaining 10% proof-of-concept. Such quasi-experimental studies are typically cross-sectional, nonrandomized, and describe the first impression of a single instant [67]. For a better understanding of the real-world effects of AI-based conversational agents on health care for chronic diseases, future research should aim at conducting field experiments, which in the best case are designed as longitudinal experimentations in order to investigate long-term effects. This is especially important when considering the time span of chronic diseases; they typically affect patients for at least 12 months but can prevail for a significantly longer period of a patient’s life span [39].

It is further noteworthy to point out that the only 2 RCTs of this review mentioned a commercial interest in the investigated conversational agent by at least one of the authors. We would encourage future research to assess commercially available conversational agents without similar business connections in order to enrich the chatbots’ evaluation by a purely external point of view.

While it is not unexpected to find that patients were the majority of targeted intervention partners, it is somewhat surprising to see that only 2 conversational agents further included additional social contacts of patients, here the patients’ parents. We want to highlight that chronic diseases often heavily affect the immediate and wider social context of the affected patient [61]. Future interventions could consider additional human involvement in order to better recognize the social effect of chronic diseases. This could further maximize treatment adherence and health outcomes, two important treatment goals [68].

Natural language processing technology is the most widely applied AI technique and outnumbers related further used techniques such as speech recognition, text-to-speech, and speech-to-text, natural language understanding, and natural language generation. Other prominent AI techniques such as deep learning, machine learning, neural networks, and decision trees are also used, but to a much smaller extent. This finding might be explained through the already mentioned prevalence of multimodal interaction approaches of the reported conversational agents, giving supremacy to the development and evaluation of communication-focused AI techniques. Currently, ongoing developments in the area of natural communication between conversational agents and humans increasingly address natural language generation and emotion recognition [69,70]. These advancements are expected to lead to AI-based conversational agents that converse even more naturally with patients than currently possible. This could have a plethora of effects on the relationship between patients and chatbots as well as on treatment-related outcomes and thus presents a relevant area for future research.

One potential danger of such presumably naturally conversing chatbots is harm or even death of the patient in case the chatbot’s recommendations are inaccurate or wrong, especially when the advice concerns critical decisions such as changes or mix of medication [71]. Patients, who are often laypeople when it comes to assessing any technical or medical capabilities of AI-based conversational agents, might follow a chatbot’s advice without additional medical clarification [71]. Future chatbot development and corresponding research should put an increased focus on addressing such shortcomings and threats in order to maximally ensure patient safety.

Except for the 2 studies developing and evaluating conversational agent architectures, the heterogeneity and general lack of depth of reported AI techniques and systems is a relevant point to consider. Even though all 10 studies explicitly state to apply AI-based systems, the lack of technical information critically hinders replicability and poses questions about the quality of reported findings. Such dearth of detail reinforces the application roadblocks of AI-based systems—opaque and biased decision-making processes and resulting lack of trust [60]. In addition, it hinders the development of a generic system architecture, which could be used as an informative framework for the development and structure of AI-based chatbots in the context of health care for chronic diseases. We strongly advise future researchers to report all necessary technical features required to replicate study results and further (partially or exemplarily) allow access to the developed AI-based conversational systems. In addition to the above-mentioned standardized guidelines for research in health care, future research should make use of already existing guidelines for reporting the technical part of AI-based conversational agents used in health care and medicine [72,73]. More generalized checklists aimed at assessing the overall structure of AI-related medical research such as the Checklist for Artificial Intelligence in Medical Imaging (CLAIM) could be also consulted; they offer guidance on which specific information should be reported on the chosen AI model and its subsequent training, evaluation, and performance [74]. We further recommend future research to synthesize a generic system architecture and derive a framework for AI-based chatbots in the context of health care for chronic diseases once the field has progressed and more standardized data are available.

Half of the studies in our review made use of external systems for the development of (parts of) their AI architecture, which could indicate a trend of external and open access–based software development for AI-based health care conversational agents. Future research should pay attention to this in order to further shed light on this approach.

A final point to consider is the inconsistent taxonomy of AI-based software; while 4 studies clearly labeled their software as AI, there was a broad variety of otherwise used terms such as natural interaction, state-of-the-art, smart, or fully automated. The inconsistent use of terms aggravates the use of a common terminology. We see value in the development and use of clear terms for the sake of clarity and comparability of future research.

### Strengths and Limitations

This systematic literature review has several strengths as well as some limitations. It was conducted and reported according to the standardized PRISMA guidelines [46]. We conducted an extensive literature search by accessing 7 databases and deploying a thorough and comprehensive search strategy. In addition, we reviewed reference lists of relevant studies and used several Google alerts containing combinations of the search terms from November 2019 until April 2020 for identifying further papers not identified through the initial database searches.

We prioritized sensitivity over specificity with our search strategy in order to avoid missing important studies and construct a holistic view of AI-based conversational agents for health care for chronic diseases. We objectively defined the study eligibility criteria. Given the novelty of the search field, however, many search results were published conference abstracts that had to be omitted given the study eligibility criteria.

Study selection, title and abstract screening, full text screening, and data extraction were done independently by two reviewers. We checked for interrater reliability at several steps in the selection process and Cohen kappa showed substantial agreement per step.

We applied a narrative approach for reviewing the included studies. Intense team discussions concerned the classification of reported AI architectures. We decided in consensus to follow the proposed taxonomy of Montenegro et al [11]. However, the final study selection might still omit relevant AI-based conversational agents if a different taxonomy for study selection were applied.

Key limitations of this review are the heterogeneity and relatively small number of the included studies as well as the prevalence of quasi-experimental studies. This underlines the complexity and novelty of the searched field, and we thus did not conduct a meta-analysis.

Finally, risk of bias varied extensively between the included studies, reducing the reliability of findings in studies with high risk of bias. This reduced the trust we could place in the reported findings of studies with high risk of bias.

### Conclusions

Technological advances facilitate the increasing use of AI-based conversational agents in health care settings. So far, this evolving field of research has a limited number of applications tailored for chronic conditions, despite their medical prevalence and economic burden to the health care systems of the 21st century. Existing applications reported in literature lack evidence-based evaluation and comparison within as well as between different chronic health conditions. Future research should focus on adhering to evaluation and reporting guidelines for technical aspects such as the underlying AI architecture as well as overall solution assessment.

## Appendix

Multimedia Appendix 1 Study protocol.

Multimedia Appendix 2 Search terms per construct.

Multimedia Appendix 3 Overview and characteristics of included studies and conversational agents.

Multimedia Appendix 4 The risk of bias tool (based upon the Consolidated Standards of Reporting Trials checklist and adapted from Maher et al [2014]).

## Abbreviations

AI: artificial intelligence
AGI: artificial general intelligence
CLAIM: Checklist for Artificial Intelligence in Medical Imaging
CONSORT: Consolidated Standards of Reporting Trials
CONSORT-EHEALTH: Consolidated Standards of Reporting Trials of electronic and mobile health apps and online telehealth
COPD: chronic obstructive pulmonary disease
JIA: juvenile idiopathic arthritis
mERA: mobile health evidence reporting and assessment
NLP: natural language processing
PRISMA: Preferred Reporting Items for Systematic Reviews and Meta-Analyses
RCT: randomized controlled trial
TREND: Transparent Reporting of Evaluations with Nonrandomized Designs
