# Supplementary material for: Artificial Intelligence-Based Conversational Agents for Chronic Conditions: Systematic Literature Review
Source: J Med Internet Res. 2020 Sep 14;22(9):e20701. doi: 10.2196/20701 (PMC7522733; doi:10.2196/20701)
Supplement: Multimedia Appendix 1 [file jmir_v22i9e20701_app1.pdf]

STUDY PROTOCOL

Schachner et al., 2020

Adopted from PRISMA-P (Preferred Reporting Items for Systematic review and Meta-Analysis Protocols) and PROSPERO

**Topic**	**Content**
Title	AI-based Conversational Agents in Chronic Healthcare: A Systematic Literature Review
Authors	Theresa Schachner, Roman Keller, Florian von Wangenheim
Review team members and their organizational affiliations	Theresa Schachner, Department of Management, Technology, and Economics, ETH Zurich, Zurich, Switzerland<br>Roman Keller, ETH Zurich, Zurich, Switzerland<br>Prof. Florian von Wangenheim, Department of Management, Technology, and Economics, ETH Zurich, Zurich, Switzerland
Contact details for of corresponding author	Theresa Schachner, tschachner@ethz.ch<br>WEV G228, Weinbergstr. 56/58, 8092 Zürich, Switzerland
Organizational affiliation of the review	Center for Digital Health Interventions, https://www.c4dhi.org/
Type and method of review	Systematic literature review
Contributions	Study design: TS; Search strategy: TS; Screening: TS, RK; Data extraction: TS, RK; Data analysis: TS; First draft: TS, RK; Revisions and subsequent drafts: TS; Critical feedback for final draft: FW
Sources/Sponsors	NA
Conflict of interest	None
Rationale	What are existing AI-based applications of Conversational Agents for chronic conditions and what are their characteristics?
Eligibility criteria	(1) Primary research studies that involved the prevention, treatment, or rehabilitation of chronic diseases<br>(2) Application of a conversational agent<br>(3) Inclusion of any kind of artificial intelligence technique such as natural language understanding or deep learning for the conversational agent architecture
Information sources	A database search will be conducted accessing PubMed Medline, EMBASE, PyscInfo, CINAHL, ACM Digital Library, ScienceDirect, and Web of Science. Search terms include synonyms, acronyms, and commonly known terms of the constructs “conversational agent”, “healthcare”, and “Artificial Intelligence”. The reference lists of relevant other literature reviews and articles will also be screened for additional relevant articles. Google alerts covering different combinations of the search term will be used to continuously retrieve additional papers until April 2020. Grey literature such as conference proceedings, posters, presentations, dissertations, and theses will be excluded.
Search strategy	Search strategy for PubMed Medline (https://pubmed.ncbi.nlm.nih.gov/)<br>Filters: none<br>Conducted in February 2020<br>“Conversational agent” OR “conversational agents” OR “conversational system” OR “conversational systems” OR “dialog system” OR “dialog systems” OR “dialogue systems” OR “dialogue system” OR “assistance technology” OR “assistance technologies” OR “relational agent” OR “relational agents” OR "chatbot" OR "chatbots" OR "digital agent" OR "digital agents" OR "digital assistant" OR "digital assistants" OR "virtual assistant" OR "virtual assistants” AND “healthcare” OR “digital healthcare” OR “digital health” OR “health” OR “mobile health” OR “mHealth” OR “mobile healthcare” AND “Artificial Intelligence” OR “AI” OR “Natural Language Processing” OR “NLP” OR “Natural Language Understanding”

	OR “NLU” OR “Machine Learning” OR “Deep Learning” OR “Neural Network” OR “Neural Networks”
Type of included study	Any type of primary research
Studied domain	Chronic conditions
Population/Participants	Any population and any participants (clinical / non-clinical, caregivers, healthcare professionals)
Data collection and selection process	Two independent reviewers will conduct the initial screening of the obtained studies based on their titles and abstracts. The same reviewers will then independently conduct full-text screening based on the eligibility/inclusion criteria. Cohen’s kappa will be calculated after each step (title screening, abstract screening, full text screening) to measure interrater reliability. Any disagreement will be discussed in person. If no consensus can be reached, the discussion will be brought to another investigator to achieve agreement.
Data items for coding	The following data items will be extracted of each included study: first author, year of publication, study location, study design/type, study aim, conversational agent evaluation measures, main reported outcomes and findings, type of chronic condition, type of study participants, name of conversational agent, goal of conversational agent, communication channel, interaction modality, AI technique, AI system development, AI categorization, funding sources, conflicts of interest. Two independent reviewers will conduct the data extraction and consensus will be reached via discussion. Any open disagreements will be resolved with a third investigator.
Outcomes and prioritization	Main outcomes: Any healthcare related intervention outcomes (e.g. type of chronic condition, health goal, intervention targets), any AI architecture related outcomes (e.g. type of AI techniques, AI system development, AI categorization)<br>Additional outcomes: Any conversational agent related outcomes (e.g. feasibility, accuracy, acceptability, functionality)
Risk of bias in individual studies	Two independent researchers will review the included studies independently to appraise their quality. Disagreement will be discussed to reach consensus. Any unresolved disagreement will be resolved with a third investigator. The application of an additional risk assessment (e.g. with the Cochrane risk bias tool or the application of risk bias assessment checklists based on the CONSORT checklist) will be discussed and applied if appropriate.
Data synthesis	The PRISMA statement will be followed for data synthesis. A narrative synthesis of the included studies will be performed.
Language	English
Country	Switzerland
Anticipated or actual start date	November 2019
Anticipated or actual end date	May 2020

Adopted from:

Booth, A., Clarke, M., Dooley, G., Ghersi, D., Moher, D., Petticrew, M., & Stewart, L. (2012). The nuts and bolts of PROSPERO: an international prospective register of systematic reviews. *Systematic reviews*, *1*(1), 2.

Moher, D., Shamseer, L., Clarke, M., Ghersi, D., Liberati, A., Petticrew, M., ... & Stewart, L. A. (2015). Preferred reporting items for systematic review and meta-analysis protocols (PRISMA-P) 2015 statement. *Systematic reviews*, *4*(1), 1.

Shamseer, L., Moher, D., Clarke, M., Ghersi, D., Liberati, A., Petticrew, M., ... & Stewart, L. A. (2015). Preferred reporting items for systematic review and meta-analysis protocols (PRISMA-P) 2015: elaboration and explanation. *Bmj*, *349*.
