# Supplementary material for: Artificial Intelligence-Based Conversational Agents for Chronic Conditions: Systematic Literature Review
Source: J Med Internet Res. 2020 Sep 14;22(9):e20701. doi: 10.2196/20701 (PMC7522733; doi:10.2196/20701)
Supplement: Multimedia Appendix 2 [file jmir_v22i9e20701_app2.pdf]

Multimedia Appendix 1: Search terms per construct

**Conversational Agent**	**Healthcare**	**Artificial Intelligence**
Conversational agent*	Healthcare	Artificial Intelligence
Conversational system*	Digital healthcare	AI
Dialog system*	Digital health	Natural Language Processing
Dialogue system*	Health	NLP
Assistance technology	Mobile health	Natural Language Understanding
Assistance technologies	mHealth	NLU
Relational agent*	Mobile healthcare	Machine Learning
Chatbot*		Deep Learning
Digital agent*		Neural Network*
Digital assistant*		
Virtual assistant*		
