# Supplementary material for: Artificial Intelligence-Based Conversational Agents for Chronic Conditions: Systematic Literature Review
Source: J Med Internet Res. 2020 Sep 14;22(9):e20701. doi: 10.2196/20701 (PMC7522733; doi:10.2196/20701)
Supplement: Multimedia Appendix 3 [file jmir_v22i9e20701_app3.pdf]

Multimedia Appendix 3: Overview and characteristics of included studies and conversational agents

Schachner et al., 2020

Abbreviations: RCT=Randomized Controlled Trial, COPD=Chronic Obstructive Pulmonary Disease, JIA=Juvenile Idiopathic Arthritis, NA=Not available, CBT=Cognitive Behavioral Therapy, NLP=Natural Language Processing, NN=Neural Networks, ML=Machine Leearning, ASR=Automatic Speech Recognition, NLU=Natural Language Understanding, NLG=Natural Language Generation, TTS=Text-to-speech, STT=Speech-to-text, AIML=Artificial Intelligence Markup Language, DL=Deep Learning, API=Application Program Interface, AI=Artificial Intelligence

General Study overview

	Study ID	Study Location	Study Design / Type	Study Aim	Conversational agent evaluation measures	Main reported outcomes and findings (extensive)
1	Ferguson *et al.* (2010)	USA	Quasi-Experimental	Design & development of prototype system	NA (previous related study: Accuracy)	-successful prototype development for data collection<br>-successful engagement of users in short daily checkup via spoken or written natural language<br>-successful development of working end-to-end spoken dialogue system for heart failure checkup
2	Rhee *et al.* (2014)	USA	Quasi-Experimental	Design & development of prototype system	-Feasability and acceptability of developed system	-Response rate for daily messages of adolescents: 81-97%<br>-Evaluation feedback: (1) Symptoms most common topic in adolscent-initated messages (2) System usage improved awareness of symptoms and triggers (3) System usage promoted treatment adherence and sense of control (4) System usage facilitated adolescent–parent partnership
3	Griol and Callejas (2016)	Spain	Quasi-Experimental	Design, development & evaluation of domain-independent framework	-Usability assessment with patients<br>-Naturalness and rehabilitative potenital assessment by caregivers	-Successful prototype development and evaluation with patients<br>-Patients' feedback: Understanding by system very good, system responses were comprehensible, adequate interaction rate, no errors perceived during interaction in tactile mode, some errors perceived in oral and multimodal mode<br>-General assessment by patients: Multimodal mode with highest overall satisfaction due to its flexibility<br>-Caregivers' feedback: Very positive assessment regarding technical aspects, perceived potential to stimulate cognitive abilities of patients, attractivity, adequateness, appropriateness, relevance
4	Ireland *et al.* (2016)	Australia	Quasi-experimental	Evaluation of chatbot	-Acceptability<br>-Functionality	-Acceptability good - Positive overall impression from first use of Harlie<br>-Functionality feedback: Technical issues with speed of processing, additional identification of problematic conevrsational responses *(not reported)*<br>-Frequent suggestion: Usage of Harlie for daily conversations without medical intent for older people in elderly care units
5	Fitzpatrick *et al.* (2017)	USA	RCT	Evaluation of fully automated conversational agent	-Feasability<br>-Acceptability<br>-Preliminary efficacy	-Chatbot interaction significantly reduced depression<br>-Chatbot interaction associated with high level of engagement; chatbot viewed as more favorably than information-only control comparison
6	Fulmer *et al.* (2018)	USA	RCT	Evaluation of fully automated conversational agent	´-Feasability	´-2 weeks of chatbot interaction with daily check-ins significantly
7	Easton *et al.* (2019)	UK	Quasi-experimental	Co-design of prototype & acceptability assessment	´-Acceptability	´-Specification of 4 distinct self-management scenarios for patient
8	Rose-Davis *et al.* (2019)	Canada	Quasi-experimental	Design, implementation & evaluation of dialogue system	-Content completeness<br>-Content relevance<br>-Content accuracy<br>-Content understanding	-Successful implementation of AI-based Extended Model of Argument into prototype conversational agent for delivering patient education<br>-Feedback: Majority of dialogue content adequat, system responses were relevant, understandable, and complete
9	Roca *et al.* (2020)	Spain	Proof-of-concept	Development & prototype architecture implementation of chatbot	-Viability / Proof of concept of architecture<br>-Input by healthcare professionals of specific chronic disease for (1) extraction of relevant disease-related parameters, (2) design and evaluation of derived disease-related functionalities	-Successful development of prototype chatbot architecture based on microservices<br>-Developed architecture implementation able to provide telemonitoring of any chronic disease through the use of messaging platforms<br>-Development and proposal of new AIML expansion called "Microservice" that enables separating chatbot conversations for different system architecture components<br>-Feedback: Developed architecture provides flexible solution for personalized monitoring services and data storage
10	Rehman *et al.* (2020)	Korea	Quasi-Experimental	Design, development & evaluation of prototype chatbot	-Overall performance measure of developed algorithm based on accuracy, precision, sensitivity, specificity, F-Measure<br>-Effectivity of voice-based user authentication<br>-User experience<br>-Efficiency	-Overall algorithm performance: Accuracy: 89%, precision: 90%, sensitivity: 89,9%, specificity: 94,9%, F-measure: 89,9%<br>-Feedback: Assessment of MIRA as "almost perfect"<br>-Effectivity of voice-based user authentication: Chatbot identifities users effectively and prevents against disguise attacks<br>-User experience: Good results in all aspects<br>-Efficiency: Chatbot efficiently predicts disease based on chief complaints and supports user in decision making

Aspect 1: Healthcare / Chronic conditions

	Study ID	Adressed Chronic Condition	Type of chronic condition	Type of study participants, Number of study participants	Type of final target interaction recipient	Health/Application goal
1	*Ferguson et al. (2010)*	Heart Failure	Cardiovascular	Heart Failure patients (focus group: n= 9, survey: n = 63)	Patients	Self-care support
2	*Rhee et al. (2014)*	Asthma	Respiratory	Adolescent asthma patient-parent dyads (n=15)	Patient-parent dyads	Self-management tool
3	*Griol and Callejas (2016)*	Alzheimer's	Psychological	Alzheimer's patients (n=25) and caregivers (n=6)	Patients	Disease monitoring
4	*Ireland et al. (2016)*	Parkinson/Dementia	Nervous system	Community members (n=33)	Patients	General conversation with Parkinson patients and facilitation of assessments, future: Speech and communication therapy for patients
5	*Fitzpatrick et al. (2017)*	Depression/Anxiety	Psychological	Students (n=70)	NA	CBT
6	*Fulmer et al. (2018)*	Depression/Anxiety	Psychological	Students (n=74)	NA	Health support via different interventions such as e.g. CBT, mindfulness-based therapy
7	*Easton et al. (2019)*	COPD	Respiratory	-Co-Design: COPD Patients (n=6), health professionals (n=5)<br>-Video-based scenario testing: COPD Patients (n=12)	Patients	Self-management tool
8	*Rose-Davis et al. (2019)*	JIA	Rheumatic	Clinicians (n=6)	Patients' parents	Patient education
9	*Roca et al. (2020)*	Variety of chronic diseases / Specific example of Psoriasis	Specific Example: Autoimmune	Healthcare professionals (n=NA)	Patients	Disease monitoring
10	*Rehman et al. (2020)*	Diabetes/Glaucoma	Autoimmune / Eye Condition	Students (n=33)	Patients	Disease diagnosis

Aspect 2: Conversational agent [CA] - Part 1

	Study ID	Conversational Agent Name	Conversational Agent Goal	Type of communication channel	Further involved humans beside patients	Dialogue initiative
1	*Ferguson et al. (2010)*	Personal Health Management Assistant	Data Collection	NA	NA	Mixed (user, proactive help by system for user)
2	*Rhee et al. (2014)*	mASMAA (mobile phone-based asthma self-management aid for adolescents)	Support	Mobile phone	-Parents<br>-Certified asthma expert via "wizard" interface (=web page to monitor interaction and intervene when necessary)	Mixed (user, system adapted to user-defined preferences)
3	*Griol and Callejas (2016)*	NA ("Application", "Conversational Agent")	Data Collection	Smartphone app (Android)	NA	User
4	*Ireland et al. (2016)*	Harlie (Human And Robot Language Interaction Experiment)	Now: Data Collection, Future: Education & Support	Smartphone app	NA	Mixed (user, system with preprogrammed random call)
5	*Fitzpatrick et al. (2017)*	Woebot	Coaching	Platform agnostic smartphone/desktop instant messenger app	NA	Mixed (user, system with one personalized message per day)
6	*Fulmer et al. (2018)*	Tess	Coaching	Customizable platform that can be accessed via multiple communication channels such as Facebook messenger, Slack, or SMS (short messaging services)	NA	NA
7	*Easton et al. (2019)*	Avachat (=avatar & chat) / Ava	Support	Web browser (using Java Script)	NA	NA
8	*Rose-Davis et al. (2019)*	JADE (Juvenile idiopathic Arthritis Dialogue-based Education)	Education	NA	Patient caregivers	User
9	*Roca et al. (2020)*	NA ("Virtual Assistant")	Diagnosis	Messaging platforms or web interfaces	NA	User
10	*Rehman et al. (2020)*	MIRA (Medical Instructed Real-Time Assistant)	Diagnosis	Platform specific application for Android; application usable on any smart Android device such as smartwatch, smartphone, tablet, laptop, and vendor-specific devices that contain a microphone, speaker, and support Android	NA	User

Aspect 2: Conversational agent [CA] - Part 2

	Study ID	Interaction modality	Input format	Output format	Availability of Conversational Agent
1	*Ferguson et al. (2010)*	Multimodal	Spoken or written natural text	Spoken or written language	NA (prototype)
2	*Rhee et al. (2014)*	Written	Written	Written	NA (prototype)
3	*Griol and Callejas (2016)*	Multimodal	Spoken, written, visual, external context (from smartphone sensors)	Multimodal (spoken, written, visual)	NA (prototype)
4	*Ireland et al. (2016)*	Multimodal	Spoken	Spoken or written language	For free on Android playstore
5	*Fitzpatrick et al. (2017)*	Written	Written	Written	Commercially available
6	*Fulmer et al. (2018)*	Written	Written natural text	Written prescripted statements	Commercially available
7	*Easton et al. (2019)*	Multimodal	Spoken or written	NA	NA (prototype)
8	*Rose-Davis et al. (2019)*	Written	NA (Written)	NA (Written)	NA (prototype)
9	*Roca et al. (2020)*	Multimodal	Written, visual (photos)	Written	NA (prototype)
10	*Rehman et al. (2020)*	Multimodal	Spoken (transcribed to written text)	Written, visual (image of map)	NA (prototype)

Aspect 3: Artificial Intelligence [AI]

	Study ID	AI techniques	Additional AI architecture details	AI system development	AI categorization
1	*Ferguson et al. (2010)*	Speech recognition, NLP	General model of collaborative problem solving as basis for agent behavior	Internal	Natural interaction
2	*Rhee et al. (2014)*	NLP	System is an extension of the existing TRIPS (The Rochester Interactive Planning System) natural system	Internal (ased on existing TRIPS (The Rochester Interactive Planning System) natural dialogue system)	State of the art
3	*Griol and Callejas (2016)*	NN, ML, ASR, NLU, NLG, TTS	-Integration of available Android speech recognition packages and Google TTS API<br>-Dialog manager integrates NLU results	External (Google API)	NA
4	*Ireland et al. (2016)*	Speech recognition incl. STT & TTS, NLP, AIML	NA	External (Google API)	AI
5	*Fitzpatrick et al. (2017)*	Decision Tree, NLP	NA	External (Woebot Labs Inc.)	Fully automated
6	*Fulmer et al. (2018)*	Emotion algorithms, ML, NLP	Implementation of ethical AI code	External (X2AI Inc.)	AI
7	*Easton et al. (2019)*	Speech recognition	NA	External (Kaldi toolkit)	AI
8	*Rose-Davis et al. (2019)*	NA	Educational content for dialogue system modelled by AI based argument theory (based on Toulmin model of argument)	Internal	AI
9	*Roca et al. (2020)*	AIML, NLP	Expansion of AIML proposed (" Microservice") to address the problem of knowing to which microservice any incoming message is directed	NA	Smart
10	*Rehman et al. (2020)*	Speech recognition, NLP, NLU, NN, ML, DL	-Speech recognition: TTS<br>-NN for signal processing	Internal	State of the art

Aspect 4: Additional study items

	Study ID	Funding stated [yes/no]	Funding yes - which?	Confict of interest stated [yes/no]	COI yes - which?	Limitations described	Reference [APA]	Journal	
1	*Ferguson et al.* (2010)	Yes	Acknowledgements: This work was supported by the Robert Wood Johnson Foundation	No	NA	NA	Ferguson, G., Quinn, J., Horwitz, C., Swift, M., Allen, J., & Galescu, L. (2010). Towards a personal health management assistant. Journal of biomedical informatics, 43(5), S13-S16.	Journal of Biomedical Informatics	.
2	*Rhee et al.* (2014)	Yes	Provost Multidisciplinary Award from the University of Rochester	No	NA	-no direct measurement of medication adherence<br>-delay in responses by wizard	Rhee, H., Allen, J., Mammen, J., & Swift, M. (2014). Mobile phone-based asthma self-management aid for adolescents (mASMAA): a feasibility study. Patient preference and adherence, 8, 63.	Patient preference and adherence	.
3	*Griol and Callejas (2016)*	No	NA	No	NA	NA	Griol, D., & Callejas, Z. (2016). Mobile conversational agents for context-aware care applications. Cognitive Computation, 8(2), 336-356.	Cognitive Computation	.
4	*Ireland et al.* (2016)	No	NA	NA	NA	NA	Ireland, D., Atay, C., Liddle, J., Bradford, D., Lee, H., Rushin, O., ... & Vogel, A. (2016). Hello Harlie: enabling speech monitoring through chat-bot conversations. In Digital Health Innovation for Consumers, Clinicians, Connectivity and Community-Selected Papers from the 24th Australian National Health Informatics Conference, HIC 2016, Melbourne, Australia, July 2016. (Vol. 227, pp. 55-60). IOS Press Ebooks.	Digital Health Innovation for Consumers, Clinicians, Connectivity and Community	.
5	*Fitzpatrick et al.* (2017)	Yes	Woebot Labs Inc. paid participant incentives	Yes	Second author is founder of the commercial entitiy Woebot Labs Inc. that created the intervention Woebot	-limited number of participants (>no mediator anaylsis possible)<br>-relatively short intervention<br>-no follow up data available whether results where sustained<br>-choice of control group was limiting (ebook not designed for multiple uses)<br>-limited generalizability due to missing socioeconomic status	Fitzpatrick, K. K., Darcy, A., & Vierhile, M. (2017). Delivering cognitive behavior therapy to young adults with symptoms of depression and anxiety using a fully automated conversational agent (Woebot): a randomized controlled trial. JMIR mental health, 4(2), e19.	JMIR Mental Health	.
6	*Fulmer et al.* (2018)	Yes	X2AI Incl. covered the cost of participant incentives	Yes	2 of the authors are employees of X2AI (creators of CA Tess) and thus have financial interest in that company	´-limited generalizabilty, esp. since socioeconomic status was not formally assessed / recruitement only via social media	Fulmer, R., Joerin, A., Gentile, B., Lakerink, L., & Rauws, M. (2018). Using psychological artificial intelligence	JMIR Mental Health	.
7	*Easton et al.* (2019)	Yes	NIHR Collaboration for Leadership in Applied Health Research and Care	No	NA	´-results limited to restricted sociodemographic sample<br>-selection bias of those who are already engaged in self management	Easton, K., Potter, S., Bec, R., Bennion, M., Christensen, H., Grindell, C., ... & Hawley, M. S. (2019). A virtual agent	JMIR	.
8	*Rose-Davis et al.* (2019)	No	NA	No	NA	-study participants from one study site only<br>-study participants familiar with used Patient Education Material (PEM)<br>-Result analysis was performed by a single coder - increases potential for bias<br>-substantial time and knowledge are required for methods<br>-coders must have substantial knowledge of material and methods and need substantial time	Rose-Davis, B., Van Woensel, W., Stringer, E., Abidi, S., & Abidi, S. S. R. (2019). Using an Artificial Intelligence-Based Argument Theory to Generate Automated Patient Education Dialogues for Families of Children with Juvenile Idiopathic Arthritis. Studies in health technology and informatics, 264, 1337-1341.	Studies in health technology and informatics	.
9	*Roca et al.* (2020)	Yes	Ministerio de Economica, Industria y Competitividad from Gobierno de Espana and European Regional Development Fund (TIN2016-76770-R and BES-2017-082017) and Gobierno de Aragon (Reference Group T31_17R) and FEDER 2014-2020 "Construyendo Europa desde Aragon"	No	NA	NA	Roca, S., Sancho, J., García, J., & Alesanco, Á. (2020). Microservice chatbot architecture for chronic patient support. Journal of Biomedical Informatics, 102, 103305.	Journal of Biomedical Informatics	.
10	*Rehman et al.* (2020)	Yes	MSIT (Ministry of Science and ICT), Korea, under the ITRC (Information Technology Research Center) support program (IITP-2017-0-01629) supervised by the IITP (Institute for Information & communications Technology Promotion), Institute for Information & communications Technology Promotion (IITP) grant funded by the Korea government (MSIT) (No. 2017-0-00655), National Research Foundation (NRF) under the NRF-2016K1A3A7A03951968 and NRF-2019R1A2C2090504	No	NA	NA	Rehman, U. U., Chang, D. J., Jung, Y., Akhtar, U., Razzaq, M. A., & Lee, S. (2020). Medical Instructed Real-Time Assistant for Patient with Glaucoma and Diabetic Conditions. Applied Sciences, 10(7), 2216.	Applied Sciences	.
