# Supplementary material for: Artificial Intelligence-Based Conversational Agents for Chronic Conditions: Systematic Literature Review
Source: J Med Internet Res. 2020 Sep 14;22(9):e20701. doi: 10.2196/20701 (PMC7522733; doi:10.2196/20701)
Supplement: Multimedia Appendix 4 [file jmir_v22i9e20701_app4.pdf]

The Risk of Bias tool (based upon the CONSORT checklist and adapted from Maher et al. (2014))

	Ferguson *et al.* (2010)	Rhee *et al.* (2014)	Griol and Callejas (2016)	Ireland *et al.* (2016)	Fitzpatrick *et al.* (2017)	Fulmer *et al.* (2018)	Easton *et al.* (2019)	Rose-Davis *et al.* (2019)	Roca *et al.* (2020)	Rehman *et al.* (2020)
**1. Title and Abstract**<br>a. identification as a randomised trial in title<br>b. structured summary	0,5	0,5	0,5	0,5	1	1	0,5	0,5	0,5	0,5
**2. Introduction**<br>a. scientific background/ rationale<br>b. specific objectives/ hypotheses	1	1	1	1	1	1	1	1	1	1
**3. Methods - trial design**<br>a. description of trial design<br>b. changes to methods after trial commencement	0,75	0,75	0,25	0,5	1	1	0,75	0,5	0	1
**4. Participants**<br>a. eligibility criteria<br>b. settings and locations of data collection	0	1	0	0,5	1	1	1	0,25	0	1
**5. Interventions**<br>Descriptions with sufficient details to allow replication	1	1	1	0,75	1	1	1	0,75	0,5	1
**6. Outcomes**<br>a. pre-specified primary and secondary outcome measures<br>b. changes to outcomes after trial commenced	0	1	1	0,75	1	1	0,75	1	0	1
**7. Sample size**<br>a. how sample size was determined<br>b. if applicable, interim analyses/ stopping guidelines	0	0,5	0,5	0,25	1	0,5	0,5	0,75	0	0,75
**8. Randomisation - *sequence generation***<br>*a. method used*<br>*b. type of randomisation including details of any restriction*	0	0	0	0	1	1	0	0	0	0,5
**9. Allocation concealment mechanism**<br>Implementation of the random allocation sequence, including concealment	0	0	0	0	1	1	0	0	0	0
**10. Implementation**<br>Who generated the random allocation sequence, who enrolled participants, and who assigned participants	0	0	0	0	0,75	0,75	0	0	0	0
**11. Blinding**<br>a. if done, who was blinded and how<br>b. if relevant, similarity of interventions	0	0	0	0	0,25	0,25	0	0	0	0
**12. Statistical methods**<br>Statistical methods used a. for primary and secondary outcomes<br>b. additional analyses	0	1	0,75	0,5	1	1	0,25	0	0	1
**13. Results - participants flow**<br>a. numbers of participants randomised, receiving treatment, and analysed<br>b. losses and exclusions, with reasons	0	0,75	0	0	1	0,5	0	0	0	0
**14. Recruitement**<br>a. dates of recruitement and follow-up<br>b. why the trial ended	0	0,5	0	0,25	1	0,5	0,5	0,75	0	0,75
**15. Baseline data**<br>A table with baseline demographic and clinical characteristics for each group	0	1	0	0,25	1	1	0,5	0	0	0
**16. Numbers analysed**<br>For each group, number of participants included in each analysis	0	1	1	0,75	1	1	0,5	0,5	0	1
**17. Outcomes and estimation**<br>a. results for each group, and the estimated effect size and its precision<br>b. absolute and relative effect sizes for binary outcomes	0	1	1	1	1	0,75	0,5	0,5	0	1
**18. Ancilliary analyses**<br>Results of any other analyses performed, distinguishing pre-specified from exploratory	0	0,75	0,5	0	0,5	0,5	0	0	0	0
**19. Harms**<br>Harms or unintended effects in each group	0	0	0	0	0	0	0	0	0	0
**20. Discussion - *Limitations***<br>Trial limitations/bias/ multiplicity of analyses	0,25	0,5	0,75	0	1	1	1	1	0,75	0,5
**21. Generalisability**<br>Generalisability (external validity, applicability) of findings	0,25	0,75	0,75	0,5	1	1	0,5	0,75	0,75	0,5
**22. Interpretation**<br>Consistent with results and balanced	0,75	1	1	0,5	1	1	1	1	1	1
**23. Other information - *Registration***<br>Registration number and name of registry	0	0	0	0	0,75	1	0	0	0	0
**24. Protocol**<br>Where the full trial protocol can be accessed	0	0	0	0	0	0	0	0	0	0
**25. Funding**<br>Sources of funding/ role of funders	1	1	0	0	1	1	1	0	1	1
**Numbers of criteria satisfied**	5,5	15	10	8	21,25	19,75	11,25	9,25	5,5	13,5
